# PANoptosis: a potential target of cardiomyopathy

**DOI:** 10.3389/fimmu.2025.1704465

**Published:** 2025-12-17

**Authors:** Mingli Sun, Changxu Lu, Jinwen Wei, Can Gao, Dan Dong

**Affiliations:** 1 College of Exercise and Health, Shenyang Sport University, Shenyang, Liaoning, China; 2 College of Basic Medical Science, China Medical University, Shenyang, Liaoning, China

**Keywords:** cardiomyopathy, PANoptosis, PANoptosome, programmed cell death, potential target

## Abstract

Cardiomyopathy is a group of heterogeneous myocardial diseases that seriously threaten patients. Because the underlying molecular pathogenesis of cardiomyopathy is still unclear, treatment options are still limited to palliative drug therapy. Hence, unraveling the molecular pathways that drive the onset and progression of cardiomyopathy is crucial for identifying effective therapeutic targets and devising clinical intervention strategies. Programmed cell death (PCD) is a type of cell death mediated by specific molecular pathways and genetically regulated, among which pyroptosis, apoptosis, and necroptosis are the main forms. Recently, researchers have uncovered that the intricate interplay among various forms of PCD has given rise to the concept of “PANoptosis,” which represents an integrated cell death process. Recent studies have found that PANoptosis is a key mediator of cardiomyopathy and is expected to become a potential therapeutic target for improving the prognosis of this disease. This review aims to summarize the current progress in understanding the association between PANoptotic activation and cardiomyopathy, and to explore new therapeutic targets and strategies for treating cardiomyopathy.

## 1 Introduction

Cardiomyopathy is classified into two types: primary and secondary. Primary cardiomyopathy originates within the heart itself; whereas secondary cardiomyopathy arises from systemic diseases or external factors. Secondary cardiomyopathy can be further subdivided based on its etiology into subtypes such as ischemic, septic, chemotherapy-induced, and diabetic cardiomyopathy (1, 2). Recent advances in cardiomyopathy classification research have facilitated diagnosis (3). However, since the underlying pathological molecular mechanisms remain unclear, current treatment is restricted to palliative pharmacological approaches or invasive therapies (4). Thus, elucidating the molecular mechanisms governing the pathogenesis and progression of this disease is important for the development of effective therapeutic targets or clinical interventions.

Programmed cell death (PCD) is a highly regulated cell death mediated by specific molecular mechanisms and plays a critical role in both physiological processes, such as tissue development, and pathological conditions, including inflammation and immune responses (5). Apoptosis, pyroptosis, and necroptosis represent three major PCD modes each characterized by distinct activation mechanisms (6–8). Early studies predominantly investigated the regulatory mechanisms underlying individual pathways. Nevertheless, accumulating evidences indicate that these pathways do not function alone but form a complicated and dynamic interaction network (9). “PANoptosis” describes a combined form of cell death that manifests hallmarks of apoptosis, pyroptosis, and necroptosis, yet defies exclusive categorization under any single one (10). In addition to being observed in the context of pathogen infection (11–13), PANoptosis has been implicated in the pathogenesis of a range of diseases, including inflammatory, cardiovascular, and neurodegenerative disorders, as well as cancers, etc. (14–16). In recent years, emerging data have highlighted the significant role of PANoptosis in cardiomyopathy and it’s potential as a therapeutic target for improving the outcomes of cardiomyopathy (17–20). However, a comprehensive description of the underlying mechanisms and therapeutic applications of PANoptosis in cardiomyopathy, is lacking.

Therefore, this review comprehensively summarizes the mechanisms of PANoptosis in different cardiomyopathy subtypes to enhance the understanding of PANoptosis-mediated pathological processes and to propose novel therapeutic targets and strategies for cardiomyopathy.

## 2 Overview of PANoptosis

### 2.1 The basis of PANoptosis

**Figure 1:**
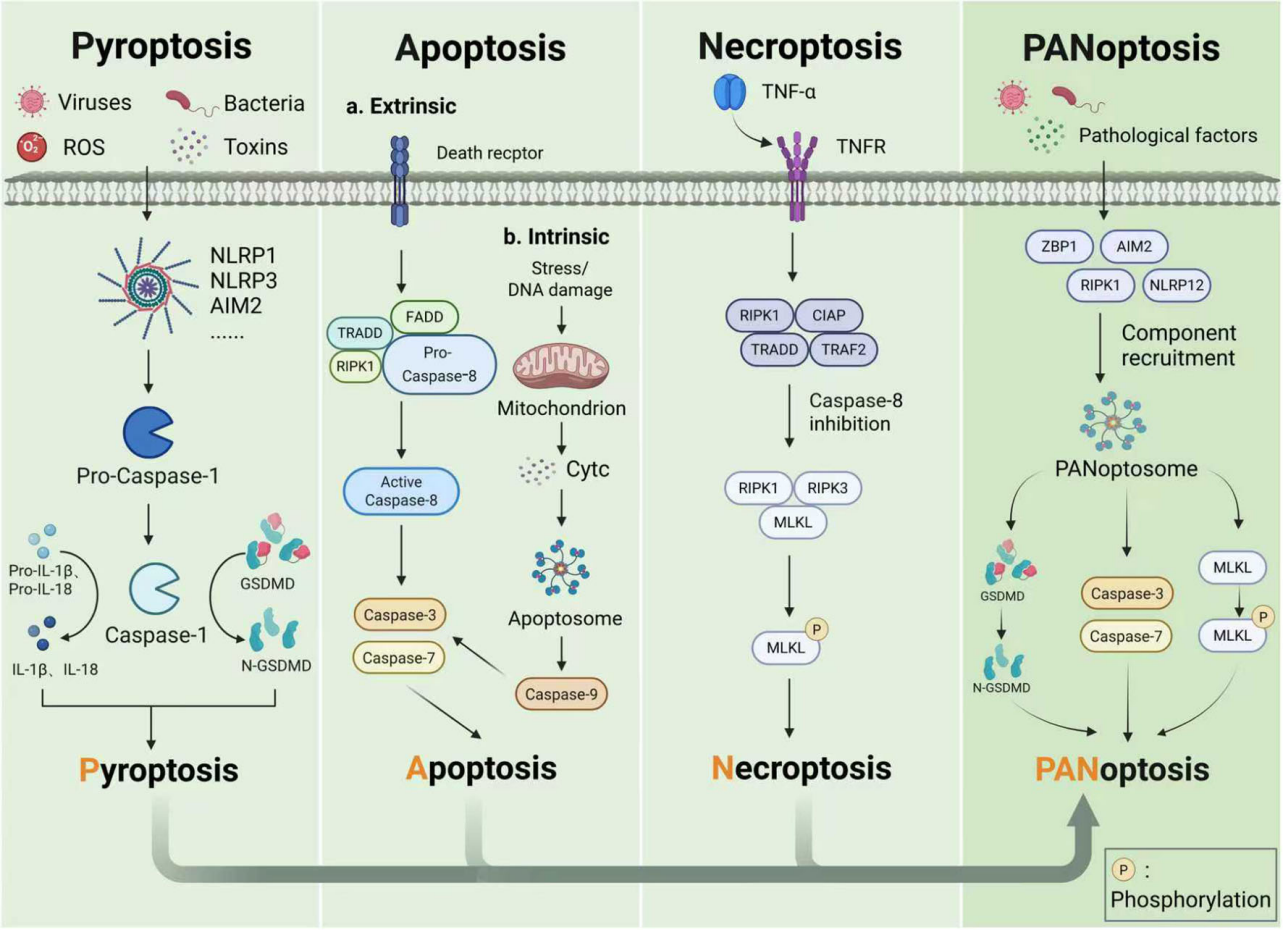
The mechanisms of pyroptosis, apoptosis, necroptosis and PANoptosis. In the canonical inflammasome-associated pyroptosis pathway, pathogen-associated molecular patterns or damage-associated molecular patterns, such as viruses, bacteria, ROS and toxins, activate inflammasomes, which subsequently lead to the activation of Caspase-1, culminating in inflammatory responses and pyroptosis. The extrinsic apoptotic pathway is mediated by death receptors and relies on the activation of Caspase-8, whereas the intrinsic apoptotic pathway is mitochondrion-dependent and involves the activation of Caspase-9. Caspase-3 and Caspase-7 serve as the terminal effectors of apoptosis. When Caspase-8 activity is inhibited, RIPK1 and RIPK3 induce necroptosis by mediating MLKL phosphorylation. PANoptosis is a programmed cell death that integrates the key features of pyroptosis, apoptosis, and necroptosis. “PANoptosis” is derived from the terms pyroptosis (P), apoptosis (A), and necroptosis (N), with “PANoptosis” denoting a form of programmed cell death. Abbreviation: ROS, Reactive oxygen species; NLRP, NLR family pyrin domain containing; AIM2, Absent in melanoma 2; CASP, Caspase; GSDMD, Gasdermin D; N-GSDMD: Gasdermin D-N-terminal domain; TRADD, Tumor necrosis factor receptor-associated death domain; FADD, Fas-associated death domain; RIPK, receptor-interacting serine/threonine protein kinases; Cytc, Cytochrome c; TNF-α, Tumor necrosis factor alpha; TNFR, Tumor necrosis factor receptor; CIAP, Calf intestinal alkaline phosphatase; TRAF2, Tumor necrosis factor receptor associated factor 2; MLKL, Mixed lineage kinase domain-like protein; ZBP1, Z-DNA-binding protein 1. The figure was created with BioRender software, ^©^ .

Pyroptosis, apoptosis, and necroptosis represent the most canonical PCD modes, providing critical protective mechanisms against both endogenous and exogenous stressors (9). As an inflammatory and immunogenic type of PCD, pyroptosis is typically initiated by inflammasome assembly and activation. A key regulatory step in this process is the activation of Caspase enzymes (21, 22). Apoptosis is a non-immunogenic cell death process that can be categorized into intrinsic and extrinsic pathways, both of which are closely associated with Caspase activation (23). Necroptosis represents a Caspase-independent, immunogenic type of programmed cell death. Its execution is primarily driven by the coordinated actions of mixed lineage kinase domain-like protein (MLKL), serine/threonine protein kinases1(RIPK1) and RIPK3 (24–26). As research into cell death continues to deepen, the interactions among pyroptosis, apoptosis, and necroptosis has gradually come to light, revealing that these three PCD pathways are not entirely independent processes. As previously discussed, both pyroptosis and apoptosis involve Caspase activation. Specifically, the activation of Caspase-1 triggers Gasdermin-D (GSDMD)-mediated pyroptosis while also exerting inhibitory effects on apoptosis (27). Furthermore, Caspase-8 not only initiates extrinsic apoptosis but also suppresses RIPK3 and MLKL-mediated necroptosis, highlighting the coordinated regulation between apoptosis and necroptosis (28). Additionally, RIPK3, a pivotal mediator of necroptosis, can activate the inflammasome and Caspase-1, thereby indicating a potential link between pyroptosis and necroptosis (29). Collectively, these findings demonstrate crosstalk among pyroptosis, apoptosis, and necroptosis. In 2019, this phenomenon was given the name “PANoptosis” (30), which represents a type of innate immune-related inflammatory PCD that incorporates crucial characteristics of pyroptosis, apoptosis, and necroptosis yet it cannot be completely accounted for by any one of these pathways alone (10). We describe the main features of PCD modes in **Figure 1**.

### 2.2 PANoptosome

PANoptosis is initiated by particular factors and modulated by the PANoptosome complex. The PANoptosome complex serves as a central hub where regulators from pyroptotic, apoptotic, and necroptotic pathways converge. By coordinating these elements, it acts as a master switch that triggers the PANoptosis. To date, multiple PANoptosome complexes featuring distinct sensors and modulators have been identified, including Z-DNA-binding protein 1 (ZBP1)-PANoptosome, absent in melanoma 2 (AIM2)-PANoptosome, RIPK1-PANoptosome, and NOD-like receptors family pyrin domain containing 12 (NLRP12)-PANoptosome.

The ZBP1-PANoptosome is primarily composed of ZBP1, RIPK1, RIPK3, Fas-associated with death domain protein (FADD), Caspase-1/6/8, NLRP3, and apoptosis-associated speck-like protein containing a CARD (ASC). This multi-protein complex was initially identified during Influenza A Virus (IAV) infection. During IAV infection, ZBP1 is activated by directly recognizing viral RNA through its ZBP1-Zα domain (31). The activated ZBP1 subsequently interacts with RIPK3 via the RHIM domain, leading to the activation of Caspase-8 and promoting MLKL phosphorylation, thereby forming the ZBP1-RIPK3-Caspase-8 complex. This process not only triggers apoptosis and necroptosis pathways but also facilitates the assembly of the PANoptosome (11, 31). ASC binds to Caspase-8 and further recruits pyroptosis-associated factors, such as NLRP3 and Caspase-1, inducing pyroptosis (32, 33). Studies have demonstrated that fibroblasts and bone marrow-derived macrophages deficient in RIPK3 do not undergo cell death upon IAV infection, which emphasizes the critical role of RIPK3 in the regulation of the ZBP1-PANoptosome (11). Additionally, Caspase-6 serves as an auxiliary protein for PANoptosome assembly by enhancing the interaction between ZBP1 and RIPK3, thereby accelerating the formation of the PANoptosome (34).

The AIM2-PANoptosome primarily include AIM2, ASC, Pyrin, and ZBP1. AIM2 serves as a cytoplasmic pattern recognition receptor and functions as a sensor for both pathogen-derived and endogenous double-stranded DNA, playing critical roles in normal human development and disease processes (35–37). Two well-characterized inducers of AIM2 inflammasome-mediated cell death are Herpes simplex virus 1 and Francisella novicida. These pathogens also serve as key inducers for the assembly of AIM2-PANoptosome and the initiation of PANoptosis (36, 38). During pathogen infection, AIM2 interacts with ZBP1 and Pyrin to form the AIM2-PANoptosome, thereby triggering PANoptosis (39). ZBP1 activates necroptosis-associated molecules RIPK3 and MLKL and collaborates with Pyrin to activate pyroptosis-related Caspase-1. Simultaneously, ZBP1 interacts with ASC, functioning as an inflammasome sensor, leading to the cleavage of Caspase-1 cleavage and triggering inflammatory cell death. ASC recruits and activates the key apoptosis mediator Caspase-8 through heterotypic interactions (39). Collectively, during pathogen infection, various cell death-related molecules orchestrate the assembly of AIM2-PANoptosome and promote PANoptosis, thereby eliciting host defense mechanisms.

The RIPK1-PANoptosome is primarily composed of RIPK1, ASC, Caspase-8, FADD, ZBP1, and NLRP3. Research has demonstrated that *Yersinia* infection can induce RIPK1-independent PANoptosis (13). During Yersinia infection, a network of interactions emerges, with RIPK1 co-immunoprecipitating key cell death regulators like ASC, RIPK3, FADD, Caspase-8, and NLRP3. This physical association strongly implicates RIPK1 in the assembly of the RIPK1-PANoptosome. *Yersinia* activates RIPK1 by inhibiting transforming growth factor β-activated kinase 1 (TAK1), thereby driving the initiation of pyroptosis, apoptosis, and necroptosis (40). However, the knockout of RIPK1 does not fully abrogate PANoptosis, indicating that compensatory mechanisms within the RIPK1-PANoptosome are triggered (40). The precise mechanisms underlying these compensatory processes warrant further investigation.

**Figure 2:**
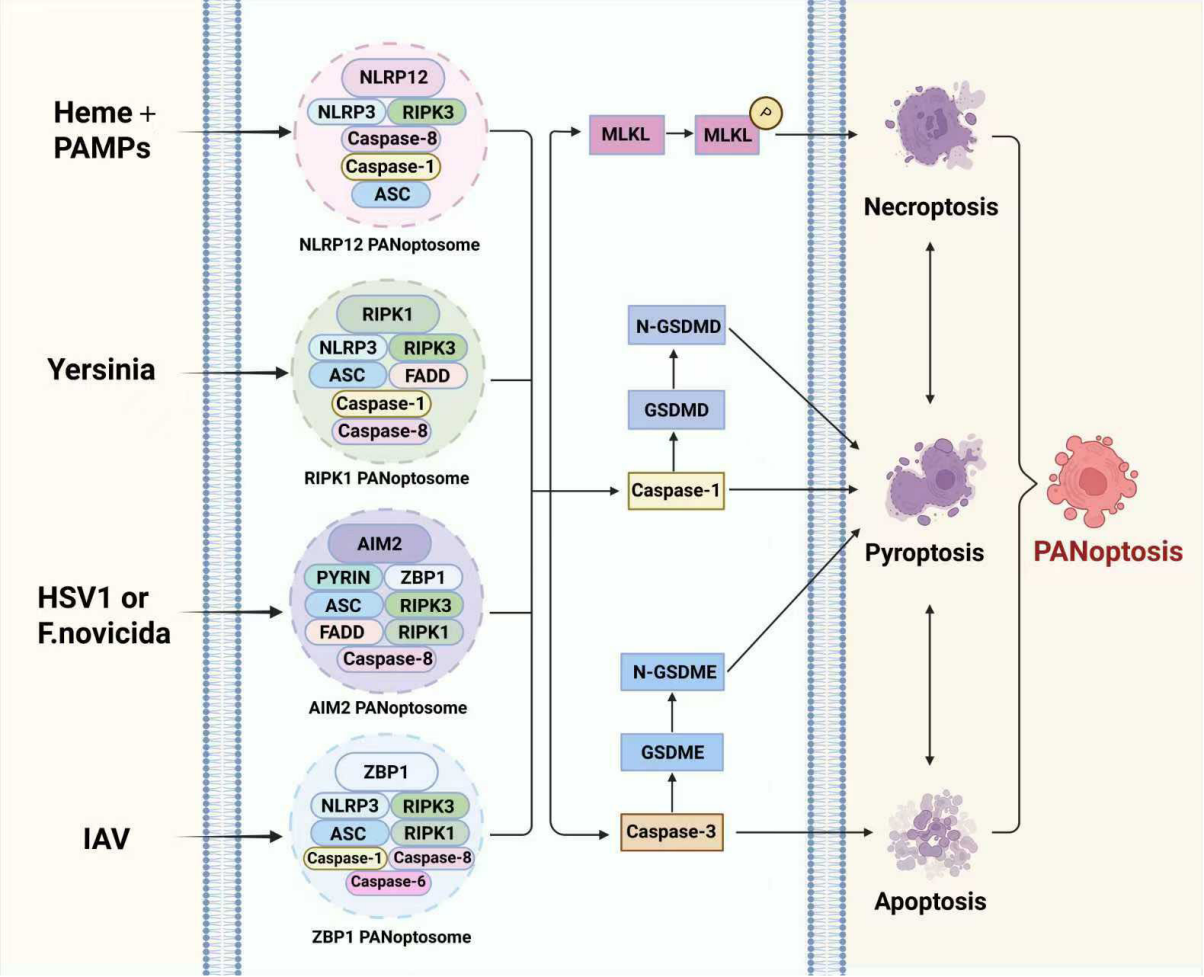
The formation mechanisms of PANoptosome. Bacterial infection and altered cellular homeostasis promote the formation of PANoptosomes, including ZBP1-PANoptosome, AIM2-PANoptosome, RIPK1-PANoptosome, and NLRP12-PANoptosome. The ZBP1-PANoptosome is primarily composed of ZBP1, NLRP3, RIPK1, RIPK3, ASC, Caspase-1, Caspase-6, and Caspase-8. The components of AIM2-PANoptosome mainly include AIM2, PYRIN, ZBP1, ASC, RIPK1, RIPK3, Caspase-8, and FADD. The components of RIPK1-PANoptosome mainly include RIPK1, RIPK3, NLRP3, ASC, FADD, Caspase-1, and Caspase-8. The NLRP12-PANoptosome is primarily composed of NLRP12, NLRP3, RIPK3, ASC, Caspase-1, and Caspase-8. These PANoptosomes further promote the activation of Caspase-3, the cleavage of GSDMD and GSDME, and the phosphorylation of MLKL, thereby ultimately contributing to the progression of PANoptosis. Abbreviation: IAV, Influenza A Virus; HSV1, Herpes simplex virus 1; PAMPs, pathogen-associated molecular patterns; ZBP1, Z-DNA-binding protein 1; AIM2, Absent in melanoma 2; TAK1, Transforming growth factor β-activated kinase 1; NLRP, NOD-like receptors family pyrin; RIPK, receptor-interacting serine/threonine protein kinase; ASC, apoptosis-associated speck-like protein containing a CARD; FADD, Fas-associated with death domain protein; GSDMD, Gasdermin-D; GSDME, Gasdermin-E; MLKL, Mixed lineage kinase domain-like protein. The figure was created with BioRender software, ^©^.

The NLRP12-PANoptosome is a newly identified PANoptosome, mainly consisting of NLRP12, ASC, RIPK3, Caspase-8, and NLRP3. Studies have demonstrated that NLRP12, functioning as a cytoplasmic innate immune sensor, can drive the activation of PANoptosomes under the stimulation of heme and pathogen-associated molecular patterns (PAMPs), thereby promoting PANoptosis (41). During this process, the upstream factor Toll-like receptor 2/4 (TLR2/4) mediates the expression of NLRP12 via the myeloid differentiation factor 88/interferon regulatory factor 1 ((MyD88/IRF1) reactive oxygen species (ROS) pathway, thereby facilitating the assembly and activation of the PANoptosome. However, the precise mechanism underlying the assembly of the NLRP12-PANoptosome remains to be elucidated. We summarize the paradigms of these PANoptosome in **Figure 2**.

### 2.3 Comparison between PANoptosis and other programmed cell death modes

The PANoptosome structurally integrates the inflammasome, apoptosis-related molecules, and core proteins of necroptosis, and its formation process is closely associated with cellular metabolic status, mitochondrial homeostasis, autophagy, and iron homeostasis (42–44). Therefore, this section aims to discuss the distinctions and interrelationships between PANoptosis and two distinct modes of PCD, autophagy and ferroptosis.

PANoptosis, is a multimodal cell death pathway, is regulated by the PANoptosome that functions to eliminate aberrant cells and initiate inflammatory responses. In contrast, autophagy is a homeostatic mechanism involving the degradation of intracellular components, which exerts dual roles in cytoprotection and non-inflammatory cell death (45). Despite marked differences in their functional roles and regulatory mechanisms, PANoptosis and autophagy may interplay in the maintenance of cellular homeostasis and the progression of disease. Clinical evidence indicates that PANoptosis-associated genes are co-upregulated with autophagy-related genes in patients with inflammatory bowel disease (46), suggesting a potential interplay between these PCDs in immune and inflammatory regulation. Furthermore, autophagy suppresses pyroptosis-related inflammatory amplification by degrading inflammasome complexes and limiting the release of IL-1β and IL-18, which are key components of the PANoptosis machinery (47–49). While autophagy generally acts as a negative regulator of PANoptosis, impaired or insufficient autophagic activity can lead to inflammasome accumulation and aggravated mitochondrial damage, thereby enhancing susceptibility to PANoptosis activation (50).

Ferroptosis is a metabolically driven form of PCD characterized by iron-dependent lipid peroxidation (51). This mechanism is distinct from PANoptosis, which integrates inflammatory, apoptotic, and necroptotic signaling pathways. Nevertheless, ferroptosis is connected with PANoptosis in mitochondrial dysfunction and ROS accumulation. Evidence indicates that in cerebral ischemia-reperfusion injury, mitochondrial impairment can simultaneously trigger both PANoptosis and ferroptosis, suggesting their potential co-activation under shared metabolic stress conditions (52). Similarly, the ferroptosis inhibitor liproxstatin-1 has been shown to ameliorate steatohepatitis within mouse models of metabolic dysfunction-associated fatty liver disease, with protective effects linked to modulation of PANoptosis pathways, highlighting a functional interplay between the ferroptosis and PANoptosis (53). Furthermore, in the tumor immune microenvironment, lipid peroxidation products and disruptions in iron homeostasis can amplify inflammatory responses by enhancing pyroptotic and necroptotic components, thereby increasing the activation probability of PANoptosis (54). In hypoxic corneal epithelial cells, lipid peroxidation scavengers have been found to attenuate both ferroptosis and PANoptosis, implicating intricate crosstalk between these cell death modalities (55). Despite growing evidence of molecular crosstalk, a deep mechanistic understanding of how ferroptosis and PANoptosis interconnect remains a major challenge in the field. In summary, comparative analysis of PANoptosis with other PCD modes not only contribute to clarify its role within the cellular fate regulatory network but also offer new perspectives for multi-targeted therapeutic strategies in disease intervention.

## 3 PANpoptosis and cardiomyopathy

### 3.1 Ischemic cardiomyopathy

Ischemic cardiomyopathy (ICM) continues to be a predominant cause of mortality on a global scale. The pathogenesis of ICM is primarily attributable to the rupture of atherosclerotic plaques, which precipitates coronary artery occlusion and culminates in persistent myocardial ischemia and irreversible tissue damage (56). While early revascularization is essential to salvage ischemic myocardium, reperfusion itself can exacerbate damage via reperfusion-triggered oxidative stress and inflammation, a process known as myocardial ischemia-reperfusion injury (MIRI) that promotes final cardiac remodeling (57, 58). Current investigations into the role of PANoptosis in ICM have predominantly centered on MIRI. Accordingly, this section delineates the established and potential mechanistic links between PANoptosis and MIRI.

The MIRI model demonstrated a concurrent upregulation of key effector molecules across PCD. The pyroptosis marker proteins GSDMD, N-GSDMD, and Caspase-1 were detected in myocardial tissues from MIRI rats and in cardiomyocytes subjected to oxygen-glucose deprivation/reperfusion (OGD/R) *in vitro*. The expression levels of necrosis markers (p-MLKL and RIPK3) and the apoptosis marker Caspase-3 were also significantly upregulated in the above experimental models. Meanwhile, the expression levels of PANoptosis-related proteins ZBP1 and Caspase-8 were also significantly increased. While inhibition of ZBP1 expression significantly reduced the levels of PANoptosis-related regulatory proteins and improved cardiac function and cardiomyocyte survival, indicating that PANoptosis plays an important role in the MIRI process (59).

The MIRI process can promote the expression of specific substances and thus activate PANoptosis. One such molecule, Arginase-1 (Arg1), is a binuclear manganese metalloenzyme that can be expressed in cardiomyocytes, vascular smooth muscle cells, and endothelial cells (60). Upregulation of Arg1 promotes ROS accumulation and cell death (61). Studies have confirmed that ischemic/hypoxic stimulation promotes lactic acid production, thereby enhancing the lactylation modification of histone H3 at lysine 18 (H3K18la) in vascular smooth muscle cells. This modification upregulates the expression of Arg1 (62). Subsequently, increased Arg1 enhances its interaction with mitochondrial inner membrane cristae structure protein 10 (Mic10), triggers structural remodeling of Mic10, and disrupts the integrity of mitochondrial cristae. This disruption promotes the release of mitochondrial DNA (mtDNA), thereby activating the cGAS-STING innate immune signaling pathway and ultimately leading to increased levels of PANoptosis-related proteins, including NLRP3, RIPK3, and MLKL (63). Another molecule, the nuclear receptor subfamily 4 group A member 1 (NR4A1) can regulate the function of the mitochondrial outer membrane protein FUN14 domain containing 1 (FUNDC1), which is involved in inducing changes in mitochondrial morphology and structure (64). It has been established that NR4A1 expression is significantly upregulated in the myocardial tissue of MIRI mice. NR4A1 regulates cardiomyocytes by phosphorylating FUNDC1 at tyrosine 18 (Tyr18), resulting in FUNDC1 inactivation and consequent inhibition of mitophagy. Furthermore, NR4A1 upregulates the expression of PANoptosis-related proteins, including ZBP1, AIM2, NLRP3, and Caspase-8, thereby aggravating MIRI injury. Conversely, cardiac-specific knockout of NR4A1 reduced the levels of these PANoptosis-related proteins, enhanced cardiomyocyte viability, and significantly alleviated MIRI injury (65). Moreover, the aberrant activation of NADPH oxidase 2 (NOX2) serves as a primary source of ROS in cardiomyocytes (66). Studies have demonstrated significant upregulation of NOX2 protein in myocardial tissues and cells from mouse models of MIRI and *in vitro* hypoxia/reoxygenation (H/R) models. This NOX2 upregulation contributes to mitochondrial oxidative stress. Concurrently, MIRI suppresses the AMPK-PGC-1α-SIRT3 signaling axis, a key pathway governing mitochondrial biogenesis. This suppression is characterized by reduced AMPK phosphorylation and decreased protein expression of PGC-1α and SIRT3, culminating in a substantial decline in mitochondrial antioxidant capacity. The resulting synergistic amplification of oxidative stress within this pathophysiological milieu promotes the marked upregulation of PANoptosis-executing proteins (e.g., RIPK3, RIPK1, GSDMD, Caspase-8), thereby triggering PANoptosis (67). In summary, during MIRI, NR4A1, Arg1 and NOX2 regulate PANoptosis by disrupting mitochondrial function.

Beyond above mitochondrial mechanism, MIRI can also activate PANoptosis by disrupting the ion channels of the cell membrane. The Piezo-type mechanosensitive ion channel component 1 (Piezo1) is a mechanosensitive ion channel crucial for maintaining normal cardiac function (68). However, its overexpression can promote the progression of cardiomyopathy. Studies have shown that Piezo1 expression is significantly upregulated in the myocardial tissue of MIRI mice and in neonatal rat cardiomyocytes subjected to hypoxia/reoxygenation (H/R) *in vitro*. This channel protein binds to Caspase-8 and further promotes the upregulation of PANoptosis-related proteins, including RIPK1, RIPK3, and MLKL. Conversely, pharmacological inhibition of Piezo1 attenuates this upregulation of PANoptosis-related proteins and thereby ameliorates MIRI-induced cardiac dysfunction (20).

In addition, MIRI can regulate PANoptosis through non-coding RNAs (ncRNAs). Studies have shown that miR-133a-3p, derived from circulating exosomes, enhances the viability of cardiomyocytes and endothelial cells and inhibits PANoptosis. Furthermore, miR-133a-3p targets and inhibits the RNA-binding protein embryonic lethal abnormal vision-like 1 (ELAVL1). While MIRI upregulates ELAVL1 expression, this protein in turn promotes NLRP3 protein expression by stabilizing NLRP3 mRNA, thereby inducing the expression of PANoptotic proteins such as GSDMD, MLKL, and RIPK3. These findings suggest that the exosomal miR-133a-3p/ELAVL1/NLRP3 signaling axis may protect cardiomyocytes and endothelial cells by modulating PANoptosis (69).

Although several findings have been obtained on the mechanism of PANoptosis induced by MIRI, the precise molecular mechanisms that govern PANoptosome assembly in MIRI remain incompletely understood. Recent studies suggest that PANoptosis during MIRI may be associated with aberrant activation of the c-Jun N-terminal kinase (JNK) signaling pathway. In a rat model of MIRI, the expression and phosphorylation levels of JNK in cardiac tissue were significantly elevated, confirming the pathway’s aberrant activation. This activation coincided with a marked upregulation of PANoptotic markers, including p-MLKL, RIPK3, Cleaved-Caspase3, and GSDMD-N. Furthermore, the assembly of the canonical ZBP1-PANoptosome characterized by increased protein levels of ZBP1, MLKL, ASC, RIPK3, Caspase-8 and NLRP3, was also observed (70). However, the upstream and downstream relationship between JNK pathway and PANoptosis and the specific assembly process of ZBP-1-PANoptosome have not been further demonstrated. Notably, another study has revealed a mechanism of ZBP1-mediated noncanonical PANoptosome assembly. Comprehensive bioinformatics analysis has suggested a critical role for ZBP1 in murine MIRI. ZBP1 expression is specifically upregulated in cardiomyocytes, particularly within the infarct zone during the reperfusion phase, with no significant localization detected in other cardiac cell types (e.g., fibroblasts, endothelial cells, or immune cells). Furthermore, in mice with cardiomyocyte-specific ZBP1 overexpression, physical interactions between ZBP1 and RIPK3, Caspase-8, and Caspase-6 were identified. In contrast, no interactions were detected with RIPK1, NLRP3, or ASC. This specific interaction profile indicates that a non-classical PANoptosome, which promotes cardiomyocyte PANoptosis, is assembled during MIRI (71). A study has reported both the upregulation of ZBP1 and the formation of a ZBP1 panoptosome with FADD and RIPK3 in a mouse MIRI model, thus suggesting that this complex may be a key mechanism for inducing PANoptosis in MIRI (59). These results collectively suggest that developing therapeutic agents which target ZBP1 itself or inhibit the subsequent assembly of the PANoptosome represents a promising strategy for suppressing cardiomyocyte PANpoptosis.

In conclusion, while MIRI constitutes an important pathophysiological mechanism in ICM, it is critical to note that MIRI is an acute, focal, and typically time-controllable injury event. It therefore constitutes only one facet of the broader and more complex pathophysiology of ICM. Future studies should further elucidate the role of PANoptosis in ICM using more comprehensive models that incorporate chronic ischemia, metabolic disturbances, and systemic factors.

### 3.2 Septic cardiomyopathy

Sepsis-induced cardiac dysfunction is clinically designated as septic cardiomyopathy (SCM) (72). Currently, the management of SCM focuses on the early recognition of sepsis, hemodynamic optimization, and etiological intervention. However, targeted therapies are still lacking (73). Therefore, a thorough elucidation of the key pathological molecular mechanisms underlying SCM is crucial for developing targeted therapeutic strategies.

Cecal ligation and puncture (CLP) and lipopolysaccharide (LPS) are established methods for inducing *in vivo* and *in vitro* models of sepsis, respectively. Studies using these models have reported a significant increase in the expression of proteins associated with pyroptosis (NLRP3, GSDMD, GSDME), necroptosis (RIPK1, RIPK3, MLKL), and apoptosis (Caspase-3, Caspase-7, Caspase-8) in septic hearts, demonstrating hallmark features of PANoptosis in SCM (74). It is noteworthy that this study observed a significant upregulation of the Piezo1 protein. To explore the potential association between Piezo1 and PANoptosis, Piezo1 inhibitors were administered. The results demonstrated that Piezo1 inhibition markedly reduced the expression of PANoptosis-related proteins in the hearts of septic mice and in neonatal mouse cardiomyocytes stimulated by LPS. Furthermore, this study suggests that the inflammatory response induced by sepsis plays a pivotal role in the upregulation of Piezo1. In an *in vitro* SCM model, the increased expression of TLR4 and the NF-κB subunit p65/RelA indicated activation of the TLR4/NF-κB signaling pathway, which was accompanied by a significant elevation in Piezo1 expression. Notably, pharmacological inhibition of NF-κB attenuated the TLR4/NF-κB-mediated induction of Piezo1 and partially improved cardiac function in septic mice. Collectively, these findings suggest that in SCM, the activation of the TLR4/NF-κB signaling pathway promotes PANoptosis through the upregulation of Piezo1. Both sepsis and I/R represent forms of acute myocardial injury. The abnormal upregulation of Piezo1 may constitute a core mechanism driving PANpoptosis in acute cardiomyopathy, though the pathway-specific activation of Piezo1 by different etiological factors remains incompletely characterized. Targeted modulation of Piezo1 could represent a potential therapeutic strategy for this cardiomyopathy subtype.

In addition to the inflammatory pathway, sepsis can also drive PANoptosis by inducing mitochondrial dysfunction. Studies have shown that LPS triggers the opening of the mitochondrial permeability transition pore (mPTP) in cardiomyocytes of septic mice, compromising the integrity of the mitochondrial inner membrane and facilitating the release of mtDNA. The accumulation of extracellular mtDNA upregulates the PANoptosis-related sensor AIM2. Subsequent binding of AIM2 to ZBP1 enhances the expression of key PANoptosome components, such as RIPK3 and Caspase-8, ultimately leading to the assembly and activation of the ZBP1 PANoptosome and the induction of PANoptosis (75). This study also found that knocking out AIM2 reversed sepsis-induced PANpoptosis and improved cardiac function in mice.

In conclusion, sepsis is a life-threatening condition characterized by a dysregulated host response to infection, resulting in systemic inflammation and multi-organ dysfunction (73). Current evidence suggests that inflammation and mitochondrial damage in cardiomyocytes serves as a central mechanism underlying sepsis-induced PANpoptosis. Consequently, cytokine inhibitors (76) and mitochondrial-targeted therapies (77) may emerge as potential treatment strategies, though further investigation and optimization are required.

### 3.3 Chemotherapy-induced cardiomyopathy

Chemotherapy-induced cardiomyopathy (CIC) has emerged as a leading cause of long-term mortality in cancer survivors. Currently, no effective strategies exist for the prevention or treatment of CIC (78). Current evidence suggests that PANoptosis may contribute to the pathogenesis of cardiomyopathy induced by both doxorubicin (DOX) and 5-Fluorouracil (5-FU).

Anthracyclines, such as DOX, are widely used chemotherapeutic agents. However, their clinical utility is hampered by the risk of inducing cumulative and dose-dependent cardiomyopathy. Furthermore, no effective strategies currently exist to prevent or treat this cardiotoxicity (79). Therefore, exploring the molecular mechanism therein may provide potential targets for DOX-induced cardiomyopathy (DIC).

Studies have shown that DOX treatment simultaneously upregulates the expression of proteins associated with multiple cell death pathways in mouse cardiac tissues. These include pyroptosis-related proteins (GSDMD, NLRP3, Caspase-1), apoptotic proteins (Caspase-3, Caspase-8), necroptotic proteins (MLKL, RIPK3), and the PANoptosis sensor ZBP1. The concomitant upregulation of these markers suggests the potential assembly of a PANoptotic complex and the concomitant activation of multiple PCD in DIC (80).

Mechanistically, some studies have revealed that DOX induces mitochondrial dysfunction by disrupting mitochondrial proteins, thereby promoting PANoptosis. Rho family GTPase 3 (Rnd3), a member of the Rnd subclass of small GTP-binding proteins, maintains mitochondrial integrity by regulating respiration and oxidative metabolism (81, 82). Following DOX injection, Rnd3 expression was significantly downregulated in the mouse myocardium, concomitant with the upregulation of markers for pyroptosis, apoptosis, and necroptosis (83). To elucidate the cardioprotective mechanism of Rnd3, this study demonstrated that it directly interacts with Rho-associated coiled-coil containing protein kinase 1 (ROCK1) in the cytoplasm. ROCK1 is a downstream effector of Ras homolog gene family member A (RhoA). Activated RhoA/ROCK1 signaling targets mitochondrial dynamin-related protein 1 (DRP1), and DRP1 phosphorylation drives excessive mitochondrial fission, thereby inducing pyroptosis, apoptosis, and necroptosis. Crucially, restoring Rnd3 expression promotes its binding to ROCK1, inhibiting ROCK1 activity and blocking the RhoA/ROCK1/DRP1 signaling pathway to inhibit PANoptosis and alleviate DIC injury. Furthermore, FUN14 domain-containing 1 (FUNDC1) is a mitochondrial receptor essential for mitophagy. It plays a crucial role in mitochondrial fission, the clearance of unfolded proteins, and the maintenance of mitochondrial homeostasis. Under normal conditions, FUNDC1 interacts with the Tu translation elongation factor (TUFM) to support the translation of proteins encoded by mtDNA (84). DOX-induced downregulation of FUNDC1 in the mouse myocardium triggers mtDNA release, which activates the cytoplasmic dsDNA sensor AIM2. The concomitant upregulation of ZBP1 and Pyrin, together with AIM2 activation, promotes AIM2-PANoptosome assembly and induces PANoptosis (18).

Beyond DOX, emerging evidence implicates the involvement of PANoptosis in the severe cardiac injury associated with another chemotherapeutic agent, 5-FU. Transcriptomic analysis in a recent study identified the p38 MAPK/JNK/ERK signaling cascade as central to 5-FU-induced cardiotoxicity in mice. This involvement was evidenced by significant upregulation and phosphorylation of the core pathway components (p38, JNK, and ERK), which occurred concurrently with elevated levels of key PANoptosis-relating proteins, including RIPK1, RIPK3, MLKL, Caspase-3, and NLRP3. This phenomenon was consistently recapitulated in H9c2 cells stimulated by 5-FU *in vitro*. Collectively, these findings suggest a close association between p38 MAPK/JNK/ERK signaling and PANoptosis activation in 5-FU stimulated cardiomyocytes. However, this study did not include intervention methods for establishing the upstream and downstream regulatory relationship between PANoptosis and the p38 MAPK/JNK/ERK signal cascade, and further clarification is still needed in the future (85).

In conclusion, currently, research on the relationship of PANoptosis and CIC primarily focuses on DOX and 5-FU, whereas investigations of other agents such as cisplatin and paclitaxel remain limited (86). These chemotherapeutic agents have been demonstrated to induce cardiomyopathy through two or more forms of PCD, suggesting the potential existence of a PANpoptotic mechanism (86–88). Further investigation is required to elucidate the PANpoptotic processes triggered by distinct chemotherapeutic agents, which will facilitate the development of targeted therapeutic interventions.

### 3.4 Diabetic cardiomyopathy

Diabetic cardiomyopathy (DCM) is a distinct cardiac manifestation in patients with diabetes mellitus (89). It is characterized by early-stage left ventricular hypertrophy and diastolic dysfunction, progressing to significant heart failure (HF) with reduced systolic function in advanced stages, in the absence of clinically significant hypertension, obstructive coronary artery disease (>50% stenosis), or moderate-to-severe valvular heart disease (90, 91). Currently, the complex phenotype of DCM remains incompletely characterized, its pathogenic molecular mechanisms remain elusive, and specific therapeutic interventions are lacking (89, 92). Therefore, by clarifying the key pathological molecular mechanisms of DCM, it will help improve the prognosis of patients with DCM.

Recent studies have shown that the expression of key PANoptosis associated proteins (including AIM2, MLKL, RIPK3, and ZBP1) is significantly upregulated in the myocardial tissue of diabetic mouse models, presenting a typical PANoptotic pattern in diabetic mouse myocardial tissue (93). This study also identified circular RNA (circRNA) as a key regulatory factor in this process. Specifically, the expression of circ-OGDH was significantly increased both in diabetic mouse models and in cardiomyocytes treated *in vitro* with high glucose and palmitic acid. Mechanistically, circ-OGDH binds to high mobility group box-1 (HMGB1) and promotes RIPK3, thereby inducing pyroptosis and apoptosis. Concurrently, it upregulates necroptosis-related proteins, collectively leading to PANoptosis.

In conclusion, there is limited research on PANpoptosis in DCM. The role PANpoptosis in DCM needs further confirmation and the formation mechanism of the PANoptosome complex in DCM remains unclear and requires further investigation.

### 3.5 Desmoplakin cardiomyopathy

Desmoplakin (DSP) is a desmosomal protein that serves as the primary force transducer between myocardial fibers and intermediate filaments (94). DSP mutations can lead to arrhythmia-induced cardiomyopathy. DSP-related cardiomyopathy is characterized by frequent left ventricular involvement, extensive fibrosis, elevated arrhythmia risk, and acute myocardial injury (95). Recent studies have found that PANoptosis is closely related to the progression of DSP-related cardiomyopathy.

A study has for the first time reported that PANoptosis is a significant pathological feature of DSP-related cardiomyopathy (17). It demonstrated that in mouse cardiomyocytes, deletion of the DSP gene leads to a post-transcriptional reduction in desmosome levels. Concomitantly, the upregulation of multiple cell death executers was observed: the apoptosis-related proteins Caspase-8 and Caspase-3; the necroptosis-related proteins RIPK1, RIPK3, and MLKL; the pyroptosis mediator GSDMD; and the PANoptosis-related protein ZBP1. From a mechanistic perspective, studies have revealed significant dysregulation of the canonical Wnt/β-catenin signaling pathway in cardiomyocytes from DSP-knockout mice. This dysregulation was manifested by the upregulation of β-catenin, its co-transcription factor T-cell factor 7-like 2 (TCF7L2), and the downstream target axis inhibition protein 2 (AXIN2). Consequently, this imbalance led to the upregulation of the apoptosis-related protein Caspase-3, the necroptosis-related protein RIPK1, and the pyroptosis executioner protein GSDMD (96). In these mice, inhibiting β-catenin was found to suppress PANoptosis, thereby improving survival rate and cardiac function, while reducing the incidence of arrhythmia and alleviating myocardial fibrosis.

In conclusion, currently, research on the role and mechanism of PANpoptosis in DSP-related cardiomyopathy remains limited, further studies are required to elucidate its association with PANpoptosis.

**Figure 3:**
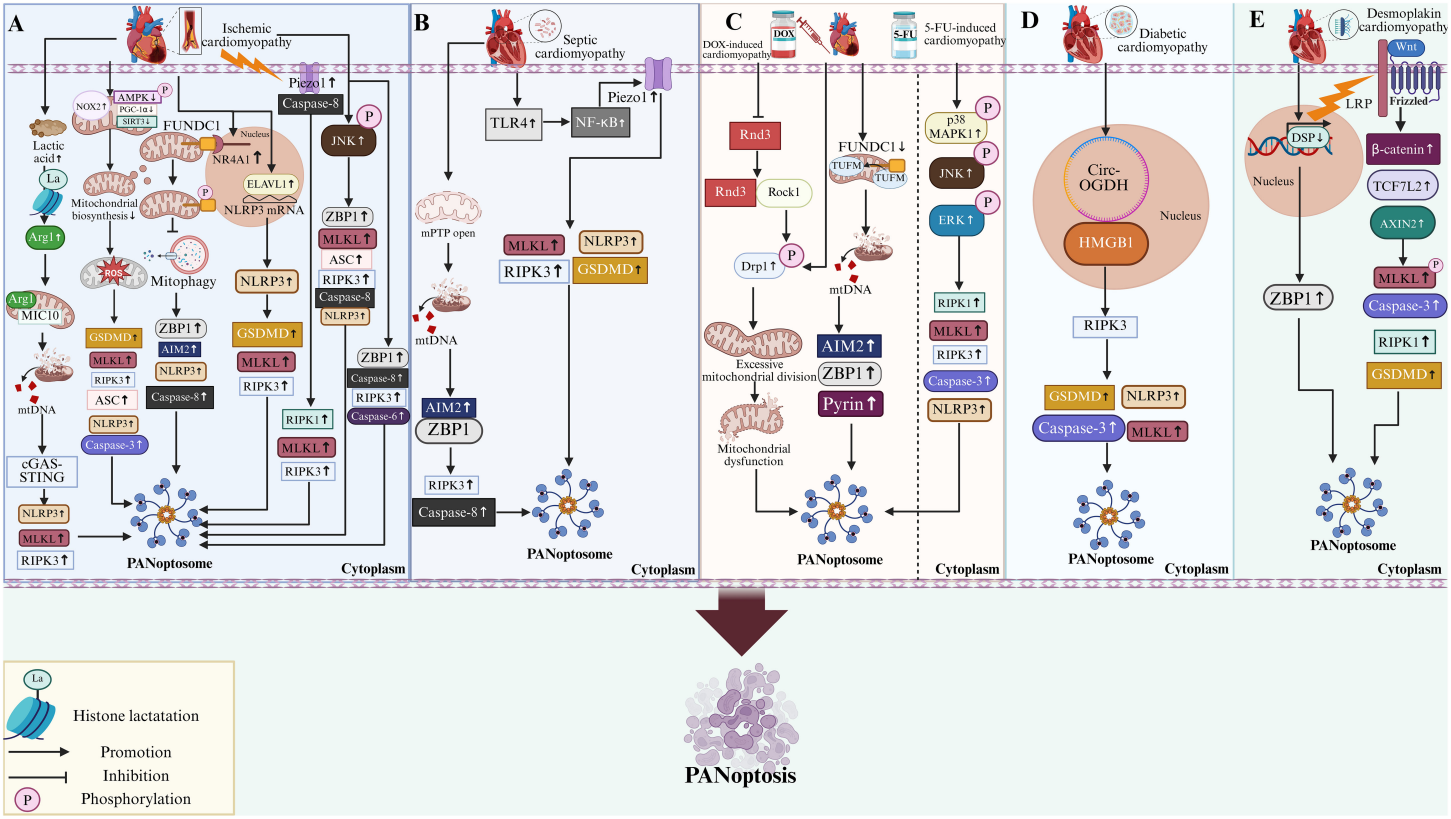
PANoptosis in cardiomyopathy. **(A)** In ischemic cardiomyopathy, hypoxia-induced lactic acid accumulation enhances histone H3 lysine 18 lactylation (H3K18la) and upregulates Arg1. Arg1 interacts with MIC10 to induce mitochondrial crista remodeling and mtDNA release. This activates the cGAS-STING signaling pathway and enhances the expression of PANoptosis-associated proteins (NLRP3, RIPK3, MLKL). Meanwhile, during ischemia-reperfusion, the expression of NR4A1 is upregulated, which impairs mitophagy through mediating phosphorylation of FUNDC1 (Tyr18) and increases the expression of PANoptosis-related proteins (ZBP1, AIM2, NLRP3, Caspase-8). Pathological conditions also elevate NOX2 protein expression and inhibit the AMPK-PGC-1α-SIRT3 mitochondrial biogenesis pathway, exacerbating mitochondrial oxidative stress and promoting PANoptosis. The upregulation of Piezo1 induced by ischemia/hypoxia promotes the binding of Piezo1 to Caspase-8, and the increase in RIPK1, RIPK3, and MLKL levels promotes PANoptosis. In addition, ischemia can also upregulate ELAVL1, which promotes NLRP3 expression by binding to its mRNA, thereby increasing the expression of PANoptosis-related proteins (GSDMD, MLKL, RIPK3). It also upregulates ZBP1, which combines with Caspase-8, Caspase-6, and RIPK3 to form a PANoptosome that drives PANoptosis. **(B)** In septic cardiomyopathy, TLR4/NF-κB activation can upregulate Piezo1, thereby increasing the expression of PANoptosis-related proteins (MLKL, NLRP3, GSDMD, RIPK3). LPS or CLP can induce the opening of mPTP, causing the release of mtDNA. The released mtDNA promotes upregulation of AIM2 expression, leading to the binding of AIM2 to ZBP1 and the upregulation of key components of the ZBP1-PANoptosome (RIPK3, Caspase-8). **(C)** In DOX-induced cardiomyopathy, DOX can inhibit the interaction between Rnd3 and Rock1 by downregulating Rnd3 expression, leading to Rock1-mediated phosphorylation of its downstream target Drp1 and promoting pathological mitochondrial fission, thereby inducing the expression of PANoptosis-related proteins. In addition, DOX treatment can downregulate the expression of FUNDC1, inhibit the FUNDC1-TUFM binding, disrupt mitochondrial structure, and trigger mtDNA release. Cytosolic mtDNA upregulates key components of the PANoptosome, such as AIM2, ZBP1, and Pyrin, promoting PANoptosis. In 5-fluorouracil (5-FU)-induced cardiomyopathy, 5-FU triggers PANoptosis by upregulating the expression and phosphorylation of p38 MAPK, JNK, and ERK, which subsequently elevates the levels of key PANoptosis marker proteins. **(D)** In diabetic cardiomyopathy, hyperglycemia upregulates circ-OGDH. circ-OGDH mediates the upregulation of PANoptosis-related proteins (GSDMD, MLKL, NLRP3, Caspase-3) by binding to HMGB1 and targeting RIPK3. **(E)** In desmoplakin cardiomyopathy, the deletion of the DSP gene leads to the upregulation of the PANoptosis-related protein ZBP1, promoting PANoptosis. In addition, dysregulation of the Wnt/β-catenin pathway (evidenced by upregulated β-catenin, TCF7L2, and AXIN2) can be observed, which in turn increases the protein expression of Caspase-3, RIPK1, MLKL, and GSDMD, and promotes PANoptosis. Abbreviation: NLRP, NOD-like receptors family; Mic10, Mitochondrial inner membrane ridge structure protein 10; AIM2, Absent in melanoma 2; ZBP1, Z-DNA-binding protein 1; LPS, Lipopolysaccharide; CLP, Cecum ligation and perforation; MLKL, Mixed lineage kinase domain-like protein; mtDNA, mitochondrial DNA; RIPK, Receptor-interacting serine/threonine protein kinase; H3K18la, Histone H3 lysine 18 lactylation; ELAVL1, Embryonic lethal abnormal vision like 1; Piezo1, Piezo-type mechanosensitive ion channel component 1; Arg-1, Arginase-1; NR4A1, Nuclear receptor subfamily 4 group A member 1; DOX, Dxorubicin; Rnd3, Rho family GTPase 3; Rock1, Rho Associated Coiled-Coil Containing Protein Kinase 1; Drp-1, dynamin-related protein 1; TUFM, Nuclear-encoded mitochondrial protein Tu translation elongation factor; TLR4, Toll-like receptors 4; NF-κB, Nuclear factor kappa B; mPTP, mitochondrial permeability transition pore; FUNDC1, FUN14 domain containing 1; circ-OGDH, circular RNA-OGDH; HMGB1, High mobility group box-1; DSP, Desmoplakin; Wnt/β-catenin, Canonical wingless-related integration/Integrated-β-catenin; TCF7L2, transcription factor 7-like 2; AXIN2, Axis inhibition protein 2. NOX2, NADPH Oxidase 2; PGC-1α, Peroxisome Proliferator-Activated receptor gamma coactivator 1 α; SIRT3, Silent information regulator 3; AMPK; Adenosine 5’-monophosphate-activated protein kinase; p38 MAPK, p38 mitogen-activated protein kinase; JNK, c-Jun N-terminal kinase; ERK, Extracellular signal-regulated kinase. The figure was created with BioRender software, ^©^.

To sum up, current research on PANoptosis in cardiomyopathy remains at an early stage. To date, investigations have primarily centered on the role and mechanism of PANoptosis in MIRI. In other forms of cardiomyopathy, the occurrence of PANoptosis is largely inferred from the concurrent dysregulated expression of key effector proteins associated with apoptosis, necroptosis, and pyroptosis. Furthermore, a major focus of existing studies is the identification of upstream mechanisms of PANoptosome within these cardiomyopathy models, aiming to uncover viable therapeutic targets. However, the precise molecular mechanisms that govern PANoptosome assembly in these cardiomyopathies remain incompletely understood. A summary of these mechanisms is presented in **Figure 3**. Additionally, the upstream events regulating PANoptosome assembly, alongside the similarities and differences in PANoptotic effector molecules among various cardiomyopathies, are summarized in **Table 1**.

**Table 1:** The PANoptosis and PANoptosome assembly in different cardiomyopathies.

Cardiomyopathy model	*In vivo*/ *In vitro*	Models (animals/cells)	Assembly of PANoptosome	PANoptosis-related genes	Pathological changes of the heart	Potential mechanisms mediating PANoptosis	Ref.
Ischemic Cardiomyopathy	*In vivo*	Male C57BL/6 mice	N/A	Caspase-3, ASC, GSDMD, NLRP3, RIPK1, RIPK3, MLKL, Caspase-8	LVEF%, LVFS%↓ CM-MB, cTNT, LDH, Myo↑	The expression of mitochondrial NOX2 was upregulated and the AMPK-PGC1α-SIRT3 pathway was inhibited	(67)
	*In vitro*	H9C2 cells			Cells viability↓		
	*In vivo*	Male C57BL/6 J mice	ZBP-1-PANoptosome	ZBP-1, RIPK3, RIPK1, Caspase-6, Caspase-8	IA/AAR, EF%, FS%↓ LDH, LVIDs↑	ZBP1 forms complexes with RIPK3, Caspase-8 and Caspase-6	(71)
	*In vitro*	Primary mouse cardiomyocytes			Cells viability↓		
	*In vivo*	Male Wistar rats	ZBP-1-PANoptosome	ZBP-1, FADD, RIPK3, Caspase-1, GSDMD, Caspase-8, MLKL, Caspase-3	LVSP, ± dp/dt_max_↓ LVEDP, CM-MB, LDH↑	ZBP1 forms complexes with FADD and RIPK3	(59)
	*In vitro*	H9c2 cells			Cells viability↓		
	*In vivo*	Sprague-Dawley rats	N/A	GSDMD, Caspase-3, ASC, NLRP3, RIPK3, RIPK1, MLKL, ZBP-1, AIM2	Infact size↓ CM-MB, cTNT, LDH↑	The JNK signaling pathway is activated	(85)
	*In vitro*	Primary rat cardiomyocytes			Cells viability↓		
	*In vivo*	C57BL/6 mice	N/A	Caspase-1, Caspase-8, NLRP3, AIM2, ZBP-1, GSDMD, Pyrin, MLKL, RIPK3, RIPK1	LVEF%↓ LVIDd, LVSd, cTNT, LDH↑	NR4A1 upregulation mediates MFF-related mitochondrial fission and FUNDC1-related mitophagy dysfunction	(65)
	*In vitro*	Primary mouse cardiomyocytes			Cells viability↓		
	*In vivo*	C57BL/6 mice	N/A	RIPK1, RIPK3, Caspase-3, Caspase-8, MLKL, NLRP3, GSDMD	EF%, FS%↓ Infact size/AAR, LDH↑	Piezo1 binds to Caspase-8 to form a complex	(20)
	*In vitro*	Neonatal rat cardiomyocytes			Cells viability↓		
Septic cardiomyopathy	*In vivo*	Male C57/BL6J mice	ZBP-1-PANoptosome	ZBP-1, MLKL, Caspase-3, Caspase-8, NLRP3	LVEF%, LVFS%↓ LVIDs, CM-MB, cTNT↑ LDH↑	Sepsis-induced inflammatory response	(19)
	*In vitro*	H9c2 cells			Cells viability↓		
	*In vivo*	Male C57BL/6N mice	N/A	NLRP3, Caspase-1, GSDMD, Caspase-8, RIPK1, RIPK3, MLKL	EF%, FS%↓ LDH↑	TLR4/NF-κB activation upregulates the expression of Piezo1	(74)
	*In vitro*	Neonatal rat cardiomyocytes/AC16 cells			Cells viability↓		
	*In vivo*	Male C57BL/6J mice	AIM2-PANoptosome	AIM2, ZBP-1, GSDMD, MLKL, Caspase-7, Caspase-8, RIPK3	LVEF%, LVFS%↓ CM-MB, cTNT↑	Mitochondrial mPTP opening leads to mt DNA efflux	(75)
	*In vitro*	Primary mouse cardiomyocytes			Cells viability↓		
Diabetic cardiomyopathy	*In vivo*	C57BL/6J mice	N/A	GSDMD, Caspase-1, MLKL, NLRP3, AIM2, ZBP1, RIPK3	EF%, FS%↓ LDH, LVIDd, LVIDs↑	Upregulation of Circular RNA-OGDH activates the HMGB1-RIPK3	(93)
	*In vitro*	H9c2 cells			Cells viability↓		
Doxorubicin- induced cardiomyopathy	*In vivo*	Male C57BL/6 mice	ZBP1-PANoptosome	NLRP3, Caspase-1, GSDMD, Caspase-8, ZBP-1, MLKL, p-MLKL, RIPK3	LVEF%, LVFS%↓ LVEDD, LVESD, CM-MB, cTNT, LDH↑	N/A	(80)
	*In vitro*	Rat’s primary cardiomyocytes			Cells viability↓		
	*In vivo*	Male WT and FUNDC1^-/-^mice	N/A	AIM-2, ZBP-1, Pyrin, Capase-1, GSDMD, Caspase-8, Caspase-3, RIPK1, RIPK3, MLKL	EF%, FS%↓	FUNDC1 inhibition causes mtDNA release	(18)
	*In vitro*	Adult mouse cardiomyocytes			Cells viability↓		
	*In vivo*	C57BL/6 mice	N/A	NLRP3, ZBP-1, AM2, Caspase-8, Caspase-3, GSDMD, Caspase-1, MLKL, RIPK3, RIPK1	LVEF%, LVFS%↓ LDH, CM-MB↑	Dysregulation of the Rnd3/Rock1/Drp1 signaling pathway	(82)
	*In vitro*	Neonatal rat cardiomyocytes			Cells viability↓		
5-fluorouracil- induced cardiomyopathy	*In vivo*	Male BALB/c mice	N/A	NLRP3, GSDMD, Caspase-1, Caspase-8, Caspase-3, RIPK1, RIPK3, MLKL	LVEF%, LVFS%↓ CM-MB, cTNT, LV Mass↑	Activation of the p38 MAPK/JNK/ERK signaling pathway	(85)
	*In vitro*	H9c2 cells			Cells viability↓		
Desmoplakin cardiomyopathy	*In vivo*	Male Dsp^F/F^mice	N/A	ZBP-1, ASC, GSDMD, RIPK1, RIPK3, MLKL, Caspase-8, Caspase-3	LVFS%↓ LVEDD, LVESD, LVMI↑	DSP Gene deletion	(17)
	*In vivo*	Male Dsp^F/F^mice	N/A	RIPK3, RIPK1, MLKL, GSDMD, ASC, Caspase-3	LVFS%↓ LVEDD, LVESD, LVMI↑	Dysregulation of the Wnt/β-catenin pathway	(96)
Polystyrene nanoplastics and cadmium jointly induced cardiomyopathy	*In vivo*	Kunming male mice	N/A	Pyrin, ZBP-1, AIM2, RIPK1, RIPK3, MLKL, GSDMD, NLRP3, Caspase-1, ASC, Caspase-8, Caspase-3	Heart weight/body weight↓ cTNT, fibrosis area↑	N/A	(111)

ASC, Apoptosis-associated speck-like protein containing a caspase recruitment domain; GSDMD, Gasdermin D; NLRP3, NOD-like receptor pyrin domain containing 3; RIPK1, Receptor-interacting serine/threonine-protein kinase 1; RIPK3, Receptor-interacting serine/threonine-protein kinase 3; MLKL, Mixed lineage kinase domain-like pseudokinase; LVEF, Left Ventricular Ejection Fraction; LVFS, Left Ventricular Fractional Shortening; CM-MB, Creatine Kinase-MB; cTNT, Cardiac Troponin T; Myo, Myoglobin; ZBP-1, Z-DNA-binding protein 1; IA/AAR, Ischemic Area/Area at Risk; EF%, Ejection Fraction; FS%, Fractional Shortening; LVIDs, Left Ventricular Internal Diameter at End-Systole; FADD, Fas-associated protein with death domain; LVSP, Left Ventricular Systolic Pressure; ± dp/dtmax, Left Ventricular Maximum Rate of Pressure Rise and Fall; LVEDP, Left Ventricular End-Diastolic Pressure; AIM2, Absent in Melanoma 2; MLKL, Mixed Lineage Kinase Domain-Like Protein; LVIDd, Left Ventricular Internal Diameter at End-Diastole; LVSd, Left Ventricular Systolic Diameter.

### 3.6 PANoptosis in pathophysiological processes of cardiomyopathy

#### 3.6.1 Pathological cardiac hypertrophy

Cardiac hypertrophy is an adaptive response to hemodynamic overload resulting from various physiological or pathological stimuli. This remodeling process is considered physiological when cardiac function is preserved, but pathological when it coincides with cardiac dysfunction (97). Pathological hypertrophy is characterized by cardiomyocyte death, resulting in decreased cardiomyocyte numbers and compensatory cellular hypertrophy, ultimately progressing to HF (98). Isoproterenol (ISO) is commonly employed to establish pathological heart failure models induced by myocardial hypertrophy (99). Studies have demonstrated significant upregulation of PANpoptosis-related proteins (NLRP3, GSDMD, Caspase-3, MLKL, RIP3, etc.) in myocardial tissue of ISO-treated mice (100). These findings indicate that PANpoptosis contributes to the pathological progression of myocardial hypertrophy. Furthermore, angiotensin II (Ang II) treatment can also induce pathological myocardial hypertrophy (101). Studies have demonstrated significant inhibition of TAK1 protein expression in Ang II-treated mouse myocardial tissue, accompanied by increased phosphorylation levels (102). As a member of the mitogen-activated protein kinase family, TAK1 typically forms stable complexes with RIPK1 (103). TAK1 inhibition or phosphorylation triggers RIPK1 dissociation, promoting PANoptosome formation and subsequent PANoptosis, ultimately leading to myocardial fibrosis and cardiac dysfunction in mice (102). Additionally, the report highlights that inflammatory infiltration (characterized by IL-1β release) represents another key factor promoting TAK1-RIPK1 dissociation, though the exact mechanism remains unclear. Future studies should investigate this aspect to better elucidate the formation mechanism of PANpoptosis in myocardial hypertrophy. Existing research primarily utilizes drug-induced models, making it difficult to exclude the direct effects of drug on PANpoptosis formation. Future studies should employ alternative modeling approaches to better elucidate the role of PANpoptosis in pathological cardiac hypertrophy.

#### 3.6.2 Heart failure

Patients with cardiomyopathy frequently exhibit signs and symptoms of HF, including dyspnea, fatigue, and edema (104). In these patients, left ventricular ejection fraction (LVEF) serves as a key parameter for assessing cardiac function. A reduced LVEF typically reflects an impairment of myocardial contractility. For decades, left ventricular ejection fraction (LVEF), as measured by echocardiography, has served as the standard parameter for diagnosing and classifying HF. According to established criteria, an LVEF ≤ 40% defines HF with reduced ejection fraction (HFrEF), an LVEF between 41% and 49% indicates HF with mildly reduced ejection fraction (HFmrEF), and an LVEF ≥ 50% defines HF with preserved ejection fraction (HFpEF) (105). PANoptosis has recently been identified as a potential mechanistic driver in the progression of HFpEF. In a mouse model of HFpEF induced by a high-fat diet and the hypertensive mimic agent L-NAME, as well as in H9c2 cardiomyocytes treated with palmitic acid and high glucose, transforming growth factor-β1 (TGF-β1) was upregulated in myocardial tissue and cardiomyocytes. Mechanistically, TGF-β1 binding to the type II TGF-β receptor (TGFBR2) on the cell membrane recruits and activates the type I receptor (TGFBR1). This activation triggers the p38 MAPK/JNK1/2 pathway, thereby promoting pathological cardiac hypertrophy. Concurrently, this signaling cascade upregulates TAK1 expression and enhances its phosphorylation. The subsequent dissociation of TAK1 from RIPK1 facilitates TAK1 activation, initiating RIPK1-mediated PANoptosis. This PANoptotic process was characterized by elevated protein levels of key mediators, including RIPK1, FADD, Caspase-8, ASC, NLRP3, and Caspase-1 (106). While programmed cell death, including pyroptosis, necroptosis, and apoptosis, has been implicated in various HF subtypes, a direct link between PANoptosis and other HF subtypes remains unestablished. Specifically, pyroptosis has been reported to promote the progression of HFmrEF and HFrEF. Furthermore, necroptosis contributes to the pathogenesis of post-infarction HF, and substantial experimental evidence supports the role of apoptosis in failing hearts (107–109). These collective findings underscore programmed cell death as a core mechanism in HF pathogenesis. Therefore, elucidating the mechanisms of PANoptosis may provide a novel rationale and foundation for targeted or adjuvant therapies for HF.

In conclusion, PANoptosis plays a significant yet distinct role across various cardiomyopathies and represents a key pathogenic mechanism driving their progression to pathological cardiac hypertrophy and HF. Consequently, targeting the PANoptosis signaling network emerges as a promising and innovative therapeutic strategy for mitigating myocardial remodeling and dysfunction.

## 4 Targeting PANoptosis in cardiomyopathy

Recent studies have demonstrated that traditional Chinese medicine formulations can effectively alleviate cardiomyopathy by regulating PANpoptosis. The Danggui Buxue Decoction (DGBXD) is a formulation consisting of Astragalus membranaceus and Angelica sinensis, both medicinal and edible herbs. Studies demonstrate that in DOX-induced cardiomyopathy models, DGBXD’s active components target and inhibit ZBP1-mediated PANpoptosis, consequently attenuating myocardial injury (80). Xiaochaihu decoction (XCHD) contains active compounds that inhibit ZBP1-mediated PANpoptosis in septic cardiomyopathy. This inhibition attenuates myocardial injury, improves cardiac function, and preserves cardiomyocyte morphology in murine models (19).

Some natural active ingredients also effectively alleviate cardiomyopathy by modulating PANpoptosis. Gypenoside I, a secondary hydrolysate of the traditional Chinese medicinal material *Gynostemma pentaphyllum*, down-regulated NOX2 protein expression while up-regulating AMPK phosphorylation and the protein levels of PGC-1α and SIRT3 in both MIRI mice and H/R-induced cardiomyocytes. This coordinated regulation restored the mitochondrial biogenesis ultimately resulted in significant inhibition of PANoptosis-executing protein upregulation (67). Tetramethylpyrazine (TMP), a bioactive compound derived from Ligusticum chuanxiong, has been demonstrated to alleviate 5-FU-induced cardiotoxicity. The compound effectively inhibits the 5-FU-induced upregulation and phosphorylation of the core pathway components (p38, JNK, and ERK) within the p38 MAPK/JNK/ERK signaling cascade. This inhibition subsequently attenuates the upregulation of PANoptosis markers, ultimately restoring cardiac function in mice (85).

Certain pharmacologic agents may ameliorate cardiomyopathy through PANpoptotic pathway inhibition. One report indicates that Penehyclidine hydrochloride (PHC), an anti-cholinergic agent, attenuates MIRI rat’s heart injury by downregulating PANoptosis-associated proteins. PHC also mitigates OGD/R-induced damage in H9c2 cardiomyocytes by inhibiting ZBP1-mediated PANoptosis signaling (59). Metformin (Met), a widely used antidiabetic drug, has been demonstrated to ameliorate cardiac function in rats with MIRI. This protective effect is mediated through the downregulation of JNK and phosphorylated JNK (p-JNK) protein levels in the myocardium, which consequently suppresses the upregulation of PANoptosis markers (70).

In addition, exogenous supplementation of essential trace elements exerts cardioprotective effects by inhibition of PANoptosis. In a mouse model of septic cardiomyopathy, exogenous Zn²^+^ supplementation prevented mPTP opening, thereby inhibiting mtDNA efflux-mediated AIM2 inflammasome activation and ZBP1-PANoptosome complex formation, ultimately attenuating PANoptosis (75).

Furthermore, exogenous administration of bioactive molecule inhibitor may similarly attenuate cardiomyopathy by preventing PANpoptosis activation. Studies indicate that in septic mouse models, the Piezo1 inhibitor GsMTx4 suppresses sepsis-induced Piezo1 upregulation, thereby reducing calcium release and preventing PANpoptosis activation, ultimately improving cardiac function (74). Furthermore, GsMTx4 markedly attenuated the I/R-mediated cardiac injury and the increase in PANoptosis-related mediators in mice (88).

**Figure 4:**
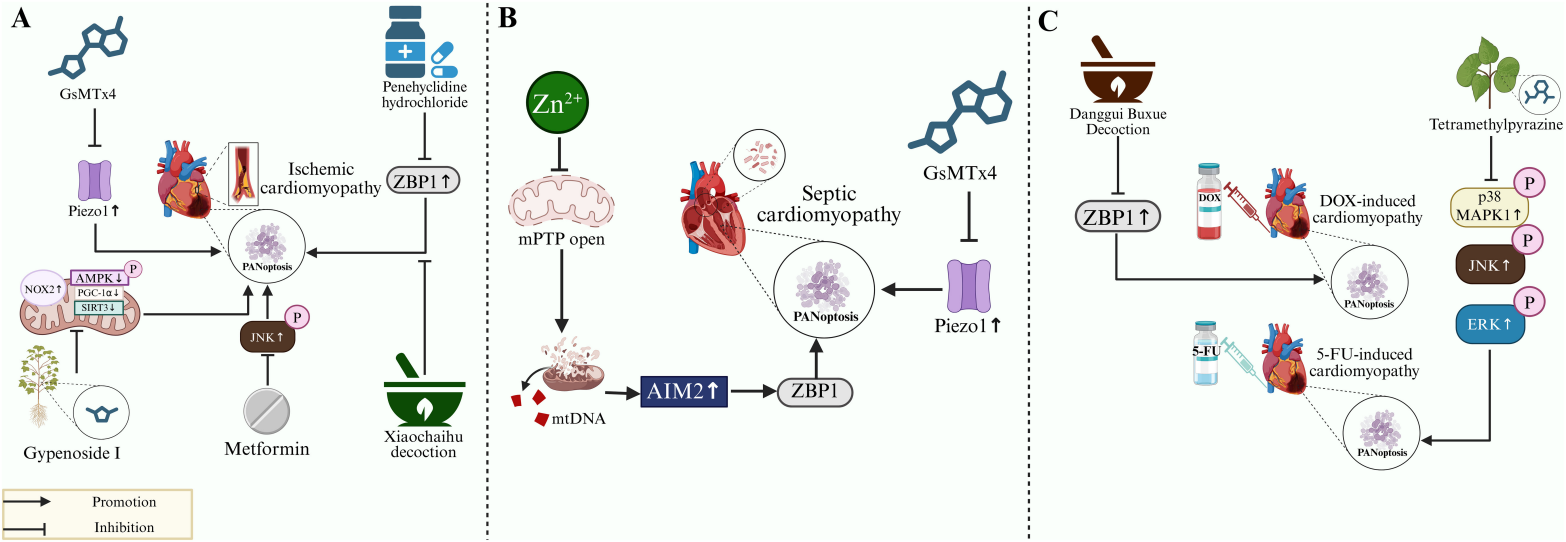
Potential therapeutic strategies for PANoptosis during cardiomyopathy. **(A)** In ischemic cardiomyopathy, the Piezo1 inhibitor GsMTx4 reduces PANoptosis through Piezo1 inhibition. Both penehyclidine hydrochloride and the Chinese herbal preparation Xiaochaihu decoction ameliorate PANoptosis via ZBP1 suppression. In addition, Gypenoside I, a hydrolysate of the Chinese herbal medicine Gynostemma pentaphyllum, significantly reduces NOX2 protein expression, enhances AMPK phosphorylation, and increases the protein levels of PGC-1α and SIRT3. This action restores mitochondrial biogenesis and inhibits PANoptosis. Furthermore, the anti-diabetic drug metformin suppresses PANoptosis by reducing both the expression and phosphorylation of JNK. **(B)** In septic cardiomyopathy models, exogenous Zn²^+^ supplementation prevents mPTP opening, thereby inhibiting AIM2 inflammasome activation (mediated by mtDNA release) and ZBP1-PANoptosome complex formation, ultimately blocking PANoptosis. GsMTx4 similarly attenuates PANoptosis by suppressing sepsis-induced Piezo1 upregulation. **(C)** In doxorubicin-induced cardiomyopathy, the active component of Danggui Buxue decoction specifically inhibits ZBP1-mediated PANoptosis. In 5-fluorouracil (5-FU)-induced cardiomyopathy, Tetramethylpyrazine (TMP) effectively inhibits the 5-FU-induced upregulation and phosphorylation of core signaling components (p38, JNK, and ERK) in the MAPK pathways; this inhibition attenuates the subsequent upregulation of key PANoptosis- associated proteins. Abbreviation: Piezo1, Piezo-type mechanosensitive ion channel component 1; ZBP1, Z-DNA-binding protein 1; mPTP, mitochondrial permeability transition pore; FUNDC1, FUN14 domain containing 1; AIM2, Absent in melanoma 2; NOX2, NADPH oxidase 2; PGC-1α, Peroxisome proliferator-activated receptor gamma coactivator 1 α; SIRT3, Silent information regulator 3; AMPK, Adenosine 5’-monophosphate-activated protein kinase; p38 MAPK, p38 mitogen-activated protein kinase; JNK, c-Jun N-terminal kinase; ERK, Extracellular signal-regulated kinase. The figure was created with BioRender software, ^©^.

In conclusion, the PANoptosome is a key component in PANoptosis targeted inhibition of any of these constituent proteins may inhibit PANoptosis and alleviate cardiomyopathy. We have summarized some substances with therapeutic potential on cardiomyopathy by regulating PANoptosis in **Figure 4**.

## 5 Summary and prospect

PANoptosis, a recently identified form of PCD, has garnered substantial attention due to its concurrent activation of apoptotic, necrotic, and pyroptotic pathways (110). Emerging evidence indicates that PANoptosis is closely associated with cardiomyopathy-related injury, with confirmed involvement in multiple myocardial pathologies including ICM, SCM, CIC, DCM, DSP-related cardiomyopathy, pathological hypertrophy and HF. However, the mechanistic relationship between PANoptosis and cardiomyopathy remains incompletely understood.

Firstly, current studies on cardiomyopathy-induced PANoptosis primarily rely on simultaneous detection of apoptosis markers (e.g., cleaved Caspase-3), pyroptosis markers (e.g., GSDMD-N), and necroptosis markers (e.g., p-MLKL) as evidence of PANoptosis activation. However, the molecular mechanisms of PANoptosome assembly and its specific regulatory processes remain uncharacterized. Additionally, other reports have identified environmental stressors such as polystyrene nanoplastic and/or heavy metal cadmium exposure that can induce myocardial injury. Studies demonstrate these exposures upregulate PANoptosis-related protein expression, triggering PANoptosis and promoting myocardial injury in mice, ultimately resulting in cardiac dysfunction (111). These findings suggest additional exogenous factors may induce PANoptosis and cause myocardial injury, warranting further investigation. Thirdly, as mentioned above inflammatory response as a key mediator of PANoptosome assembly and PANoptosis across these cardiomyopathy subtypes, immunomodulatory strategies may be a promising therapeutic avenue for cardiomyopathy. However, this requires further investigation. Moreover, when systematically administered (e.g., intraperitoneally or orally), traditional Chinese medicine prescriptions, conventional drugs, and natural compounds frequently exhibit poor water solubility, inadequate organ targeting, and low bioavailability (112, 113). Nanomedicine-based delivery systems could overcome these limitations by enabling precise organ-specific targeting (114). For instance, a study demonstrated that a tannic acid (TA)- and N-acetyl-L-cysteine (NAC)-protected bimetallic cluster nanoenzyme can target the mouse heart and effectively reduce DOX-induced cardiomyocyte PANoptosis (115). In addition to nanomedicine-based delivery systems, studies have demonstrated that miRNA-based interventions can be delivered via circulating exosomes (116). This approach effectively inhibited ELAVL1-NLRP3 binding by delivering miR-133a-3p to myocardial ELAVL1 through extracellular vesicles, thereby suppressing I/R-induced PANpoptosis in mouse cardiomyocytes (69). These methods may provide novel therapeutic strategies for cardiomyopathy; however, clinical research in this area still requires further development. Furthermore, beyond pharmacological interventions, non-pharmacological strategies like exercise training represent a promising approach to reducing PANoptosis and ameliorating cardiomyopathy. Exercise training has demonstrated efficacy in alleviating pathological manifestations in various cardiomyopathies, including DCM (117), ICM (118), DSP-related cardiomyopathy (119) and DIC (120). Furthermore, the protective effects of exercise training are linked to the attenuation of cardiomyocyte death processes, including apoptosis, pyroptosis, and necroptosis, across various rodent models of cardiomyopathy (121–123). However, whether exercise training mitigates PANoptosis in cardiomyopathy models requires further investigation.

In summary, PANoptosis significantly contributes to the pathogenesis of cardiomyopathy and has attracted considerable research attention in recent years. This suggests that current understanding of PANoptosis in cardiomyopathy remains preliminary, with substantial gaps in both mechanistic knowledge and clinical translation. Further elucidation of its molecular mechanisms and associated signaling networks is essential for developing more effective and safer therapeutic strategies for cardiomyopathy.
